# A highly sensitive *in vivo* footprinting technique for condition-dependent identification of *cis* elements

**DOI:** 10.1093/nar/gkt883

**Published:** 2013-10-03

**Authors:** Rita Gorsche, Birgit Jovanovic, Loreta Gudynaite-Savitch, Robert L. Mach, Astrid R. Mach-Aigner

**Affiliations:** ^1^Research Division Biotechnology and Microbiology, Institute of Chemical Engineering, Vienna University of Technology, Gumpendorfer Str. 1 a, A-1060 Vienna, Austria and ^2^Department of Biology, University of Ottawa, Gendron Hall, 30 Marie Curie, Ottawa, ON, K1N6N5, Canada

## Abstract

Knowing which regions of a gene are targeted by transcription factors during induction or repression is essential for understanding the mechanisms responsible for regulation. Therefore, we re-designed the traditional *in vivo* footprinting method to obtain a highly sensitive technique, which allows identification of the *cis* elements involved in condition-dependent gene regulation. Data obtained through DMS methylation, HCl DNA cleavage and optimized ligation-mediated PCR using fluorescent labelling followed by capillary gel electrophoresis are analysed by ivFAST. In this work we have developed this command line-based program, which is designed to ensure automated and fast data processing and visualization. The new method facilitates a quantitative, high-throughput approach because it enables the comparison of any number of *in vivo* footprinting results from different conditions (e.g. inducing, repressing, de-repressing) to one another by employing an internal standard. For validation of the method the well-studied upstream regulatory region of the *Trichoderma reesei xyn1* (endoxylanase 1) gene was used. Applying the new method we could identify the motives involved in condition-dependent regulation of the *cbh2* (cellobiohydrolase 2) and *xyn2* (endoxylanase 2) genes.

## INTRODUCTION

The sequence-specific binding of transcription factors to the DNA is a key element of transcriptional regulation (1–3). Therefore, the knowledge of which areas of an upstream regulatory region (URR) are specifically targeted by proteins is essential for the further understanding of regulatory mechanisms. For this purpose *in vivo* and *in vitro* footprinting methods employing nucleases such as DNaseI (4–7) or alkylating agents such as dimethylsulfate (DMS) (8,9) are routinely used to detect protein–DNA interactions. DMS treatment of DNA leads to methylation of guanine and adenine residues, with each guanine or adenine residue of purified DNA having the same probability of being methylated. When used for *in vivo* footprinting DMS readily penetrates living cells. There, protein–DNA interactions cause either a decreased accessibility of certain G or A residues to DMS (protection) or an increased reactivity (hypersensitivity) (10).

The URRs of eukaryotic DNA are complex and include a number of different recognition sites that can be targeted by multiple transcription factors at a time (2). Furthermore, the important regulatory elements are often hundreds of bases away from the transcription start (1), necessitating the coverage of large regions in the footprinting reactions. Additionally, various genes and transcription factors are grouped together in regulons. Elucidating the binding characteristics of transcription factors as well as the transcriptional regulation and interdependencies in regulons requires the analysis of footprinting patterns of the URRs of a number of different genes under various different conditions. Therefore, a standardized, high-throughput approach to traditional *in vivo* footprinting allowing parallel investigation of a number of conditions and/or isolates is necessary.

The original protocol for DMS *in vivo* footprinting was already established in 1985 (8,9) and has been improved upon since then by adding ligation-mediated PCR (LM-PCR) (11). LM-PCR quantitatively maps single-strand DNA breaks having phosphorylated 5′-ends within single-copy DNA sequences. Briefly, it involves blunt-end ligation of an asymmetric double-stranded linker onto the 5′-end of each, before cleaved, blunt-ended DNA molecule. This linker adds a common and known sequence to all 5′-ends allowing exponential PCR amplification of an adjacent, unknown genomic sequence (12). Furthermore, optimizing the polymerase and cycling conditions (13), and adapting the method to different kinds of cells, from cell lines (8,11,14,15) and yeast (9) to filamentous fungi (16), was achieved. Nevertheless, due to the use of polyacrylamide gels and radioactive labelling of the DNA fragments the resulting protocol was laborious, used hazardous substances, yielded results of strongly varying quality, and consequently, was not yet suitable for high-throughput projects.

The use of fluorescent labels and separation of DNA fragments by capillary sequencer has meanwhile been introduced to a number of similar techniques, such as RFLP (17), AFLP (18), *in vitro* DNaseI footprinting (19) or chromatin analysis (20,21). In 2000, an approach applying automated LM-PCR with infrared fluorochrome-labelled primers and a LI-COR DNA sequencer for detection was used to compare *in vivo* to *in vitro* UV-treated DNA (22). In this study we employed [6-FAM]-labelling of the DNA fragments in DMS *in vivo* footprinting and analysis via capillary sequencer employing an internal size standard. Moreover, we made use of analysis by a certified sequencing service, which guarantees stable and controlled analysis conditions. This resulted in a fast and sensitive way to analyse fragment size as well as peak intensities in a large number of samples, providing an excellent tool for comparison of URRs in a number of different isolates and different conditions. The final step to an automated high-throughput *in vivo* footprinting technique is the manner in which the acquired data is processed. Traditional *in vivo* footprinting employs visual comparison to align sequences with band patterns and densitometric measurements to determine band intensities [e.g. (11,23–25)]. For standardized comparison of multiple samples from different experiments, a computational processing of the analysis data is paramount. Therefore, we developed a data analysis tool (termed ivFAST) that plots normalized peak area ratios against sequence data and automatically determines which bases are protected from or hypersensitive to methylation by DMS.

To test the new method we examined part of the Xyr1/Cre1 regulon of *Trichoderma reesei* (teleomorph *Hypocrea jecorina*). *Trichoderma reesei* is a filamentous ascomycete of great industrial importance because of its high potency in secretion of hydrolases. Xyr1 is recognized as the essential activator for most hydrolytic-enzyme encoding genes in *T. reesei*, e.g. *cbh1*, *cbh2* (Cellobiohydrolases I and II-encoding) and *egl1* (Enoglucanase I-encoding), as well as *xyn1* and *xyn2* (Xylanases I and II-encoding) (26,27). Previous footprinting experiments identified a 5′-GGC(T/A)_3_-3′-motif as the Xyr1-binding site in the URRs of *cbh2*, *xyn1*, *xyn2* and *xyn3* (28–31). Cre1, on the other hand, is characterized as a repressor responsible for mediating carbon catabolite repression of hydrolytic-enzyme encoding genes (32), such as *cbh1* and *xyn1* (33,34). 5′-SYGGRG-3′ was found to be the consensus sequence for Cre1-binding (35).

In this study, the URR of the above-mentioned *xyn1* gene was used to validate the method. By using traditional, gel-based *in vivo* footprinting next to the new, software-based method we found that the new method allows not only a comparison of *in vivo* methylated samples to naked DNA (i.e. *in vitro* methylated, genomic DNA used as a reference), but is sensitive enough for a comparison of *in vivo* methylated samples with each other. This we demonstrate by applying the new method to the URRs of the *cbh2* and *xyn2* gene. These URRs are of similar architecture bearing the so-called cellulase-activating element [CAE; 5′-ATTGGGTAATA-3′; (31)] or xylanase-activating element [XAE; 5′-GGGTAAATTGG-3′; (30)], respectively, of which both were previously identified as essential for gene regulation. By employing the new method we have detected the following motifs: (i) the CAE and the XAE, (ii) other generally known, but in these URRs so far unrecognized motifs (such as Xyr1- or Cre1-binding sites) and (iii) so far unknown motifs.

## MATERIALS AND METHODS

### Strains and growth conditions

The ascomycete *H. jecorina* (*T. reesei*) QM9414 [ATCC 26921; a cellulase hyper-producing mutant derived from wild-type strain QM6a (36)] and an according *xyr1* deletion strain (23) were used in this study and were maintained on malt agar. For replacement experiments mycelia were pre-cultured in 1-l-Erlenmeyer flasks on a rotary shaker (180 rpm) at 30°C for 18 h in 250 ml of Mandels-Andreotti (MA) medium (37) supplemented with 1% (w/v) glycerol as sole carbon source. An amount of 10^9^ conidia per litre (final concentration) were used as inoculum. Pre-grown mycelia were washed and equal amounts were re-suspended in 20 ml of MA media containing 1% (w/v) glucose, 0.5 mM d-xylose, 1.5 mM sophorose as sole carbon source or no carbon source, respectively, and incubated for 3 h (growth conditions) or 5 h (resting cell conditions). For *in vitro* DNA methylation mycelium grown on rich medium (3% malt extract, 1% glucose, 1% peptone) was used.

### *In vivo* methylation of genomic DNA

Methylation of DNA *in vivo* was performed according to Wolschek *et al.* (16). An amount of 40 µl of DMS in 2 ml MES (200 mM, pH 5.5) were added to 20 ml of fungal culture and incubated at 30°C and 180 rpm for 2 min. Methylation was stopped with 100 ml of ice-cold TLEβ buffer [10 mM Tris pH 8, 1 mM EDTA, 300 mM LiCl, 2% (v/v) β-mercaptoethanol]. Mycelia were harvested, washed with TLEβ buffer and distilled water, and frozen in liquid nitrogen. DNA extraction was performed according to standard protocol (38). The DNA was cleaved at methylated purines by incubating 100 µl of DNA solution (∼100 µg) with 6.3 µl HCl (0.5 M) on ice for 1.5 h (39). The DNA was precipitated with 25 µl sodium acetate (3 M, pH 5) and 500 µl ethanol, dissolved in 250 µl bi-distilled water and incubated at 90°C for 30 min with 10 µl NaOH (1 M). After addition of 25 µl Tris (1 M, pH 7.5) and adjustment of the pH to 7.5, the DNA fragments were again precipitated with sodium acetate and ethanol, dissolved in 100 µl Tris (10 mM, pH 7.5) and purified using the QIAquick Nucleotide Removal Kit (Qiagen, Hilden, Germany).

### *In vitro* methylation of genomic DNA

For *in vitro* methylation genomic DNA extracted from mycelium grown on full medium was methylated according to Mueller *et al.* (14). An amount of 100 µl of DNA solution (∼100 µg) was incubated with 400 µl of DMS reaction buffer (0.05 M sodium cacodylate, 0.001 M EDTA, pH 8) and 2 µl of DMS (1:20 dilution in bi-distilled water) at room temperature for 5 min. The reaction was stopped by adding 50 µl of stop solution (1.5 M sodium acetate, 1 M β-mercaptoethanol). The DNA was precipitated twice with sodium acetate and ethanol and dissolved in 100 µl Tris (10 mM, pH 7.5). Cleavage of the DNA was performed as described above. This DNA was used as one reference and we refer to it using the term ‘naked DNA’ throughout the manuscript.

### Traditional, gel-based analysis of DNA fragments via LM-PCR

LM-PCR was performed using Vent Polymerase [New England Biolabs (NEB), Ipswich, MA] as described by Garrity and Wold (13). End-labelling of RG72-2 using γ-^32^P-ATP was done according to Mueller and Wold (11) and resulting DNA fragments were extracted with phenol/chloroform/isoamylalcohol (25:24:1, vol/vol) and precipitated with ethanol. The DNA pellet was re-suspended in 10 µl of loading dye (0.05% bromophenol blue, 0.05% xylene cyanol, 20 mM EDTA), heated at 95°C for 5 min and loaded on a 6% polyacrylamide sequencing gel.

### Generation of DNA fragments via modified LM-PCR

LM-PCR was modified from the original protocol of Mueller and Wold (11) and the adaptation of Wolschek *et al.* (16). First-strand synthesis was performed in a 30 µl reaction mixture containing 1× buffer (NEB), 0.01 µM oligo 1, 0.2 mM dNTPs, 1 U Vent polymerase (NEB) and 300–400 ng DNA template. The following PCR program was performed: denaturation at 95°C for 5 min, annealing at 55.5°C for 30 min and elongation at 75°C for 10 min. For the annealing of the linker oligonucleotides 21 µmol each of oligo-long and oligo-short in 400 µl of Tris (0.25 M, pH 7.7) were heated at 95 °C for 5 min and slowly cooled to 30°C (0.01°C/s). For ligation of the linker the sample was put on ice and 4 µl of T4 ligase buffer [10×, Promega Corporation (PC), Madison, WI, USA], 4 µl of linker and 1.5 U of T4 DNA ligase (Promega) were added. After incubation at 17°C overnight the DNA fragments were precipitated with sodium acetate, ethanol and 10 µg of tRNA, and dissolved in 10 µl of Tris (10 mM pH 7.5).

Amplification of the DNA fragments was performed in a 25 µl reaction mixture containing 10 µl sample DNA, 1× buffer (NEB), 0.2 mM dNTPs, 0.2 µM oligo 2, 0.2 µM oligo-long, and 1 U Vent polymerase (NEB). The PCR program was the following: initial denaturation at 95°C for 2.5 min followed by 17 cycles of 1 min at 95°C, 2 min at 60.5°C and 3 min at 75°C.

For the labelling reaction 1 U of Vent polymerase (NEB) and oligo 3 (5′-[6-FAM]-labelled, 0.2 µM final concentration) were added and the following PCR program was performed: initial denaturation at 95°C for 2.5 min, followed by five cycles of 1 min at 95°C, 2 min at 63.5°C and 3 min at 75°C.

All LM-PCR reactions were performed in triplicates.

### Separation of 6-FAM-labelled DNA fragments

Separation of the fluorescently labelled DNA-fragments via capillary gel electrophoresis (CGE) was performed by Microsynth AG (Balgach, Switzerland) on an ABI 3730 XL Genetic Analyser (Life Technologies Corporation, Carlsbad, CA, USA) using GeneScan™ 600-LIZ as internal size standard (Life Technologies). Data from DNA fragment analysis, i.e. peak area values and DNA fragment length, was determined using Peak Scanner™ Software v1.0 (Life Technologies).

### Analysis of peak data

To improve sample throughput the analysis of CGE data were automated using ivFAST (*in vivo* footprinting analysis software tool). This software tool was developed and used for the first time during this work. It is a command line-based program, written in Java 6. For the heatmap creation the JHeatChart library (http://www.javaheatmap.com/) was used. This is a Java library for generating heatmap charts for output as image files, which is open source under an LGPL license (http://www.gnu.org/licenses/lgpl-3.0.en.html). ivFAST reads in plain text files containing the CGE results from a specified folder, as well as a DNA sequence file in FASTA format. Given a start point in the DNA sequence and a direction, the program maps the measured peaks to the given sequence and removes background peaks not matching an A or G in the sequence (according to the default setting). The peak area of valid peaks is normalized against total peak area and the share of standard peaks in total peak area to account for variance in the CGE analysis. In addition, normalization against the ratio of unincorporated primers to total peak area is used to account for differences in PCR efficiency. From sample replicates (at least duplicates) the mean peak area and the sample variance (based on a Student’s distribution) is calculated for each peak. To determine whether peaks differ significantly from sample to sample their 95% confidence intervals (two-sided) for the mean of the sample replicates are checked to be non-overlapping (pairwise comparison of samples). If this criterion is fulfilled, the quotient of the mean peak areas of sample to reference sample is calculated. From the result of this calculation a text file as well as a heatmap is created, where protected bases with quotients <1 are printed in three shades of red and hypersensitive bases with quotients >1 are printed in three shades of blue. The ivFAST manual, which explains how the software works and how to use it, is included in the software package. From there, the step-by-step conversion of the data, the according algorithms and the normalization of data can be inferred in all details.

A minimum of two replicates needs to be available to run the software. The authors recommend using (at least) three replicates, which was done throughout this study.

### RNA-extraction and reverse transcription

Harvested mycelia were homogenized in 1 ml of peqGOLD TriFast DNA/RNA/protein purification system reagent (PEQLAB Biotechnologie, Erlangen, Germany) using a FastPrep FP120 BIO101 ThermoSavant cell disrupter (Qbiogene, Carlsbad, USA). RNA was isolated according to the manufacturer’s instructions, and the concentration was measured using the NanoDrop 1000 (Thermo Scientific, Waltham, USA).

After treatment with DNase I (Fermentas, part of Thermo Fisher Scientific, St. Leon-Rot, Germany), synthesis of cDNA from 0.45 µg mRNA was carried out using the RevertAid™ H Minus First Strand cDNA Synthesis Kit (Fermentas); all reactions were performed according to the manufacturer’s instructions.

### Quantitative PCR analysis

All quantitative PCRs (qPCRs) were performed in a Rotor-Gene Q cycler (QIAGEN). All reactions were performed in triplicate. The amplification mixture (final volume 15 µl) contained 7.5 µl 2× ABsolute™ QPCR SYBR® Green Mix (ABgene, part of Thermo Fisher Scientific, Cambridge, UK), 100 nM forward and reverse primer and 2.0 µl cDNA (diluted 1:100). Primer sequences are provided in Table 1. Each run included a template-free control and an amplification-inhibited control (0.015% SDS added to the reaction mixture). The cycling conditions were comprised of a 15 min initial polymerase activation at 95°C, followed by 40 cycles of 15 s at 95°C, 15 s at 60°C (*xyn2, xyr1* and *act*) and 15 s at 72°C; for *sar1*, following the initial activation/denaturation, we ran 40 cycles of 15 s at 95°C and 120 s at 64°C. All PCR efficiencies were >90%. Data analysis, using *sar1* and *act* as reference genes, and calculations using REST 2009 were performed as published previously (40).

**Table 1.** Oligonucleotides used in this study

Name	Sequence 5′– 3′	Usage
RG53	GAATTCAGATC	*iv*-FP, oligo-short
RG54	GCGGTGACCCGGGAGATCTGAATTC	*iv*-FP, oligo-long
RG67	AAGTCATTGCACTCCAAGGC	*iv*-FP, *xyn1* oligo 1 fw
RG68	CCTCTTCACATCATGATTTGAGC	*iv*-FP, *xyn1* oligo 1 rev
RG69	ATTCTGCAGCAAATGGCCTCAAGCAAC	*iv*-FP, *xyn1* oligo 2 fw
RG70	CAAGTGAGGTTGAAAGCGGCTCGTA	*iv*-FP, *xyn1* oligo 2 rev
RG71	[6-FAM]CTGCAGCAAATGGCCTCAAGCAACTACG	*iv*-FP, *xyn1* oligo 3 fw
RG72	[6-FAM]GAGGTTGAAAGCGGCTCGTACAGTATCC	*iv*-FP, *xyn1* oligo 3 rev
RG72-2	GAGGTTGAAAGCGGCTCGTACAGTATCC	*iv*-FP, *xyn1* oligo 3 rev
RG97	AAGCGCTAATGTGGACAGGATT	*iv*-FP, *cbh2* oligo 1 fw
RG98	CAATACACAGAGGGTGATCTTAC	*iv*-FP, *cbh2* oligo 1 rev
RG99	CATTAGCCTCAAGTAGAGCCTATTTCCTC	*iv*-FP, *cbh2* oligo 2 fw
RG100	GCCTCTTCAGGTGAGCTGCTG	*iv*-FP, *cbh2* oligo 2 rev
RG101	[6-FAM]GCCTCAAGTAGAGCCTATTTCCTCGCC	*iv*-FP, *cbh2* oligo 3 fw
RG102	[6-FAM]CTTCAGGTGAGCTGCTGTGAGACCATG	*iv*-FP, *cbh2* oligo 3 rev
RG127	GTTCCGATATATGAGATTGCCAAG	*iv*-FP, *xyn2* oligo 1 fw
RG128	GTTGATGTCTTCTTGCTTCAGC	*iv*-FP, *xyn2* oligo 1 rev
RG129	AGCCGTTATTCAGACAATGTATGTGCCG	*iv*-FP, *xyn2* oligo 2 fw
RG130	GGAGTTGTTGTGTCTTTTGGGCTTGG	*iv*-FP, *xyn2* oligo 2 rev
RG131	[6-FAM]CCGTTATTCAGACAATGTATGTGCCGGGC	*iv*-FP, *xyn2* oligo 3 fw
RG132	[6-FAM]GTTGTTGTGTCTTTTGGGCTTGGAGGGG	*iv*-FP, *xyn2* oligo 3 rev
act fw	TGAGAGCGGTGGTATCCACG	*act* qPCR
act rev	GGTACCACCAGACATGACAATGTTG	*act* qPCR
sar1 fw	TGGATCGTCAACTGGTTCTACGA	*sar1* qPCR
sar1 rev	GCATGTGTAGCAACGTGGTCTTT	*sar1* qPCR
cbh2 fw	CTATGCCGGACAGTTTGTGGTG	*cbh2* qPCR
cbh2 rev	GTCAGGCTCAATAACCAGGAGG	*cbh2* qPCR
xyn1 fw	CAGCTATTCGCCTTCCAACAC	*xyn1* qPCR
xyn1 rev	CAAAGTTGATGGGAGCAGAAG	*xyn1* qPCR
xyn2 fw	GGTCCAACTCGGGCAACTTT	*xyn2* qPCR
xyn2 rev	CCGAGAAGTTGATGACCTTGTTC	*xyn2* qPCR

## RESULTS AND DISCUSSION

### Development of an improved, software-based *in vivo* footprinting technique

#### Motivation for method design

Improving the original *in vivo* footprinting protocol was necessary for a number of reasons. Besides the fact that switching from radioactive to fluorescent labelling is preferable for safety reasons, detection of labelled DNA fragments by CGE instead of densitometric analysis of a sequence gel is significantly faster, more accurate and more sensitive, especially since the use of a commercial sequencing service ensures stability and reproducibility of the fragment length analysis. A further goal of the method improvement was to permit the analysis of a large sample set simultaneously, as well as to enable comparisons of samples based on varying reference samples. Finally, an increase in sensitivity compared with the original protocol was anticipated.

#### Method description and optimization

**Figure 1.**
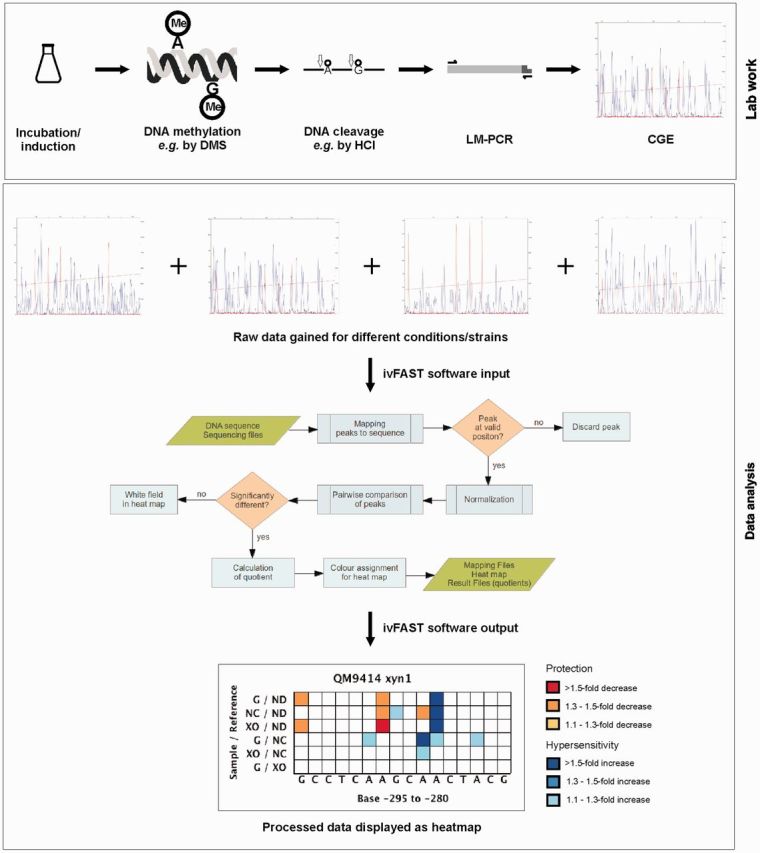
Schematic presentation of the workflow and generation of final data. The main steps of the software-based, high-throughput *in vivo* footprinting method comprise growing/incubating the microorganism under conditions to be investigated (e.g. inducing conditions), *in vivo* DNA methylation using e.g. DMS, DNA extraction, DNA cleavage by e.g. HCl followed by LM-PCR and CGE. A subset of CGE analyses results to be compared (raw data) are submitted to electronic data analysis using the ivFAST software for generation of the results displayed as final heatmap (processed data output). The steps of processing the data by the ivFAST software can be inferred from the flowchart (for more details see the ivFAST manual). Heatmap: *x*-axis gives the analysed DNA sequence; *y*-axis shows which samples are referred to each other (e.g. G/ND means ‘glucose repressing conditions referred to naked DNA’); only signals that are statistically different are considered; protected bases are highlighted in red shades and hypersensitive bases are highlighted in blue shades; 1.1- to 1.3-fold difference between compared conditions is shown in light shaded colour, 1.3- to 1.5-fold difference between compared conditions is shown in middle shaded colour and >1.5-fold difference between compared conditions is shown in dark shaded colour.

The main steps of the procedure are depicted in Figure 1. First, fungal mycelia were incubated under different cultivation conditions of interest (inducing, repressing, de-repressing). The *in vivo* methylation of fungal mycelia was performed as described before using DMS (16). DNaseI cannot enter the fungal cell and therefore, was not used for *in vivo* footprinting in this study. DNA extraction of genomic DNA was followed by DNA cleavage using HCl, which led to DNA breaks at methylated guanine and adenine residues. Next, LM-PCR was applied because it is a sensitive and specific technique for visualization of *in vivo* footprints. To determine the optimal number of cycles for the amplification and labelling reaction in the LM-PCR, reactions with 17 and 20 cycles for the amplification step, and 5, 10, 15 and 20 cycles for the labelling reaction were conducted. Samples obtained by *in vivo* methylation and subsequent extraction and cleavage of genomic DNA from fungal mycelia (*in vivo* methylated samples) as well as *in vitro* methylated, fungal genomic DNA (naked DNA) as a reference were used as templates. For the amplification step 20 cycles turned out to be too many, because even though differences in peak area values between naked DNA and *in vivo* methylated samples could be detected, *in vivo* methylated samples from different cultivation conditions did not show any significant differences (data not shown). This suggested that the reaction had already reached the end of the exponential phase and the concentrations of DNA fragments had started to level. When stopping the reaction after 17 cycles clear differences between samples from different cultivation conditions can be detected (data not shown), consequently it was chosen. As for the labelling reaction, samples with five and 10 cycles showed an increase in peak area values, while the peak area values did not increase for 15 and 20 cycles (data not shown), indicating that fewer cycles are sufficient to produce clear fluorescence signals. A comparison of reactions with five and 10 cycles again showed that an increase in cycles resulted in a decrease in distinction of different cultivation conditions (data not shown). Consequently, five cycles were chosen as optimal for the labelling reaction.

#### Development of ivFAST

Performing footprinting reactions of large sample sets simultaneously requires a software-based data analysis. Therefore, in this work we developed a software tool to facilitate data analysis. First, the peak area values and DNA fragment lengths are extracted from the *.fsa-files received from the custom service after CGE (e.g. Supplementary Figure S1) to plain text files. The essential steps of the data analysis are incorporated into a command line-based program: i.e. plotting against the DNA sequence, normalization of peak area values and filtering statistically significantly different bases (protected or hypersensitive) according to a chosen reference sample (compare flowchart in Figure 1). This software tool is easy to use and permits analysis of a dataset and visualization of the results in a very short time, i.e. data analysis starting from obtained CGE results can be done in 10 min per sample (given that three replicates are used). ivFAST is freely available at http://www.vt.tuwien.ac.at/ biotechnology_and_microbiology/gene_technology/mach_ aigner_lab/EN/. From there, both the software and a detailed manual can be downloaded. The manual explains how to use the software and how it works including the step-by-step conversion of the data, the according algorithms and the normalization of data. On the one hand, ivFAST actually determines the precise intensity of protection or hypersensitivity and yields as output the exact number given in a text file. On the other hand, ivFAST also displays results in a gradual mode of visualization (three shades for each, protection and hypersensitivity, of which the range is manually adjustable) and yields as output a heatmap as *.png-file for graphic display of results.

### Validation of the newly developed *in vivo* footprinting technique

#### Comparison of the new technique to traditional *in vivo* footprinting

**Figure 2.**
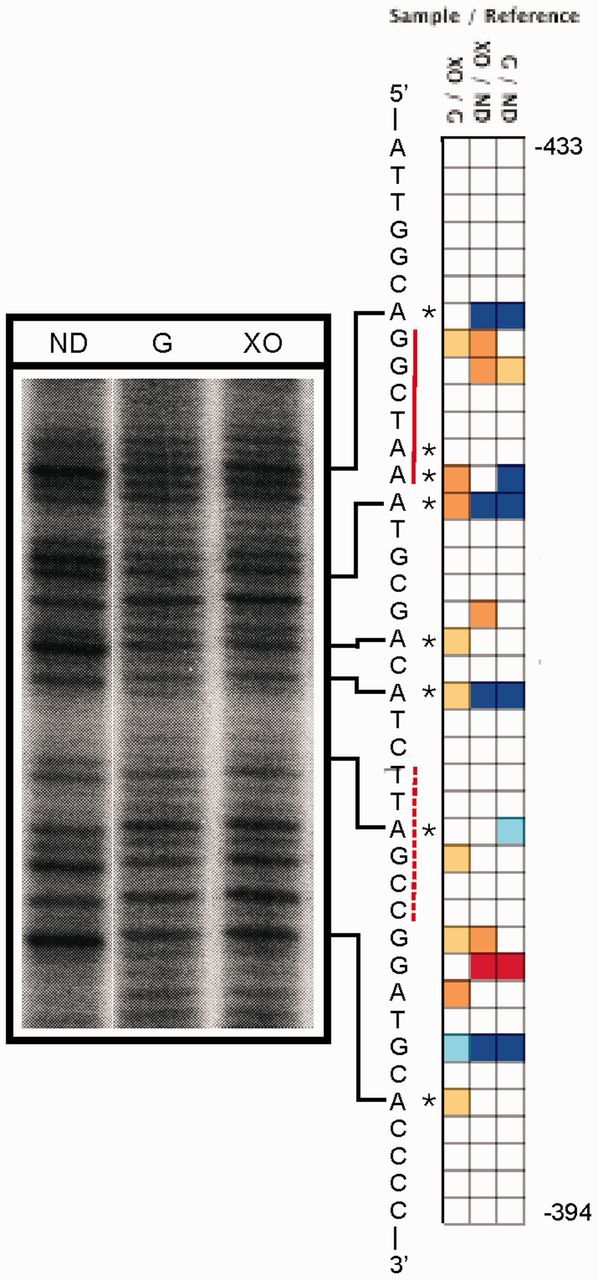
Comparison of the traditional *in vivo* footprinting to the newly developed method. *In vivo* footprinting analysis of the coding strand of a *xyn1* URR (−433- to −394-bp upstream from ATG) covering two Xyr1-binding sites, which are indicated by red lines (solid, site is located on the coding strand; dashed, site is located on the non-coding strand), was performed. *Trichoderma reesei* cultivated on glucose (G) or d-xylose (XO) followed by DMS-induced *in vivo* methylation and naked DNA as a reference (ND) were analysed. Left side shows a gel obtained by the traditional method. Asterisks indicate protected bases. Right side shows a heatmap yielded by the newly developed method.

As a first attempt, the newly developed, software-based technique was compared with the traditional, gel-based *in vivo* footprinting approach. Because the URR of *xyn1* is well-studied and the *cis* elements involved and the contacting *trans* factors are widely known, it was chosen for a comparative investigation of both techniques side-by-side. As Xyr1 is the main transactivator of the *xyn1* gene expression, an URR part covering two Xyr1-binding sites [previously proven functional by deletion analysis (34,41)] was analysed. Using traditional *in vivo* footprinting, the protection of some bases could only be detected when compared with naked DNA, whereas no condition-specific differences (regardless if repressing or inducing) were found (Figure 2). In contrast, the new technique generally yielded more protection/hypersensitivity signals compared with the gel if samples from *in vivo* footprinting were compared with naked DNA (Figure 2, G/ND, XO/ND). Most strikingly, the new technique also displays signals if *in vivo* footprinting results from inducing conditions (d-xylose) were compared with those from repressing conditions (glucose) (Figure 2, XO/G). Summarizing, the traditional, gel-based method and the comparison to naked DNA applying the new method revealed a similar *in vivo* footprinting pattern under repressing and inducing conditions. However, only the new method detects clear induction-specific differences, which are in good accordance with *xyn1* transcript data (Supplementary Figure S2a).

#### Reproducibility of the new technique

**Figure 3.**
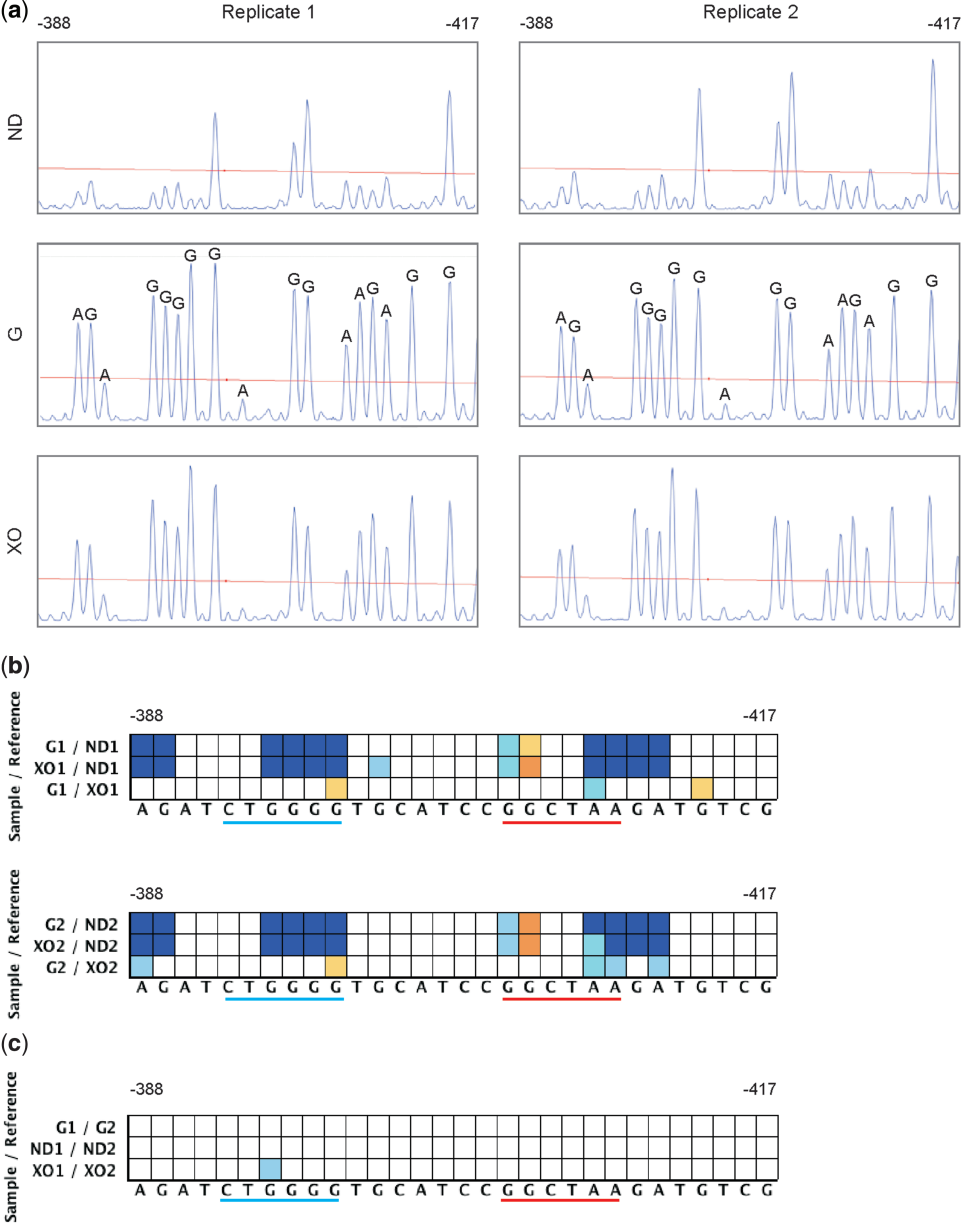
Comparison of two biological replicates analysed by the newly developed *in vivo* footprinting method. *In vivo* footprinting analysis of the non-coding strand of a *xyn1* URR (−388- to −417-bp upstream from ATG) covering a Cre1-binding site (underlined in blue) and a Xyr1-binding site (underlined in red) was performed. *Trichoderma reesei* cultivated on glucose (G) or d-xylose (XO) followed by DMS-induced *in vivo* methylation and naked DNA as a reference (ND) were analysed. (**a**) Original data of two biological replicates (Replicates 1 and 2) obtained after CGE displayed as electropherograms next to each other. Peaks in the electropherograms of the glucose replicates are marked by the corresponding DNA bases for easier orientation. (**b**) Analysed data of two biological replicates (indicated by the numbers 1 and 2) using ivFAST displayed as heatmaps under each other. (**c**) Analysed data of two biological replicates (indicated by the numbers 1 and 2) using ivFAST, if one replicate refers to the other, displayed as a heatmap.

In order to test the reproducibility of the method, *in vivo* footprinting of samples from two different conditions (repressing and inducing) and from two biological replicates of each was performed. The original trace data of these samples and—as a reference—of naked DNA (performed also in duplicates) is pictured in Figure 3a. Comparing the electropherograms of the replicates, it becomes clear that their peak pattern is the same. The peak pattern of the naked DNA strongly differs from both types of *in vivo* footprinting samples (repressing/inducing condition). If the *in vivo* footprinting sample from repressing conditions (glucose) is compared with the one from inducing conditions (d-xylose), slight differences in certain peak ratios can be observed. These findings support the above-mentioned conclusion that strong differences can be detected comparing *in vivo* footprinting samples with naked DNA, but also detection of condition-dependent differences (comparing *in vivo* footprinting results from a certain condition to another) are now possible. Using these raw data for analysis applying ivFAST, a heatmap for each replicate is obtained (Figure 3b). They yield a similar signal pattern, regardless if the *in vivo* footprinting samples are referred to naked DNA (Figure 3b; compare G1/ND1 and XO1/ND1 with G2/ND2 and XO2/ND2) or to each other (Figure 3b; compare G1/XO1 with G2/XO2). Most importantly, the heatmap that results from referring the same type of replicate (glucose, d-xylose, naked DNA) to each other is given (Figure 3c). As expected hardly any signal is yielded in this case supporting a sufficient reproducibility of the method.

#### Verification of signals yielded by the new technique

**Figure 4.**
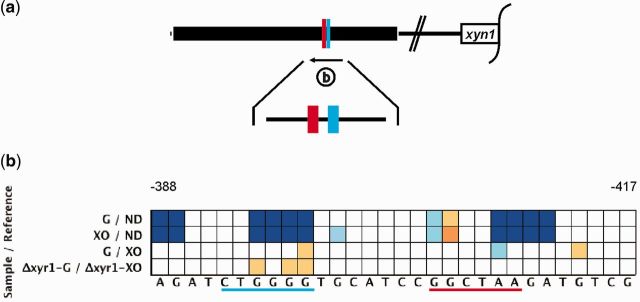
Comparison of *in vivo* footprinting results of a deletion and a parental strain. (**a**) *In vivo* footprinting analysis of a *xyn1* URR (−388- to −417-bp upstream from ATG) covering a Cre1-binding site (underlined in blue) and a Xyr1-binding site (underlined in red) was performed. (**b**) The non-coding strand was analysed after incubation of the *T. reesei* parental and a *xyr1* deletion strain (Δxyr1) on glucose (G) or d-xylose (XO) followed by DMS-induced *in vivo* methylation. Naked DNA was used as a reference (ND).

In order to test the reliability of the signal yielded by the new technique, we used the wild-type and an isogenic *xyr1* deletion strain for *in vivo* footprinting analyses. Xyr1 is the main transactivator of *xyn1* gene expression (26). Consequently, a region of the *xyn1* URR covering a functional binding site for Xyr1 was chosen for investigation. The consensus sequence for Xyr1 DNA binding [5′-GGC(A/T)_3_-3′] was previously investigated by EMSA and *in vitro* footprinting (28). As a control, the investigated region also includes a functional binding site for another transcription factor involved in *xyn1* gene regulation, namely Cre1 (34), which is still intact (Figure 4a). The consensus sequence for Cre1 DNA binding (5′-SYGGRG-3′) was previously investigated by EMSA and *in vitro* footprinting (35). As before, the strains were cultivated on glucose (repressing condition) or d-xylose (inducing condition). As mentioned above, again, reference to naked DNA generally highlights a high number of bases as protected or hypersensitive, but does not provide a condition-specific pattern (Figure 4b). A direct comparison of *in vivo* footprinting results (repressing conditions referred to inducing conditions) of the wild-type (Figure 4b, G/XO) with those of the *xyr1* deletion strain (Figure 4b, Δxyr1-G/Δxyr1-XO) revealed that while the hypersensitivity at the Xyr1-binding site disappears, the protection at the Cre1-binding site is increased in the deletion strain. This observation is not unexpected as the activator Xyr1 is not contacting this regulatory region in the deletion strain, and Cre1, which was shown to be involved in chromatin packaging (42), can now deploy its repressor function unrestrainedly.

### Applying the new *in vivo* footprinting technique to previously identified URRs

#### *In vivo* footprinting of the URR of the *cbh2* gene

**Figure 5.**
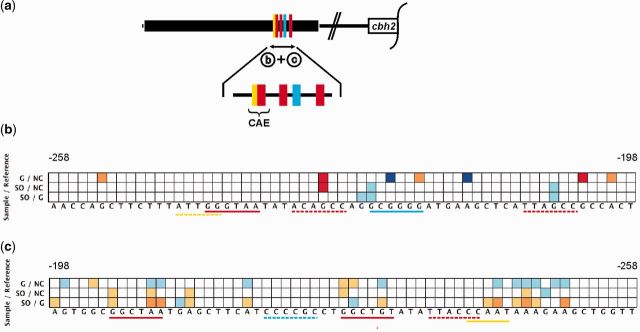
*In vivo* footprinting analysis of the *T. reesei cbh2* URR. (**a**) A *cbh2* URR covering the cellulase activating element (CAE) comprising a CCAAT-box (yellow) and a Xyr1-binding site (red), two additional Xyr1-binding sites and a Cre1-binding site (blue) was investigated (−258- to −198-bp upstream from ATG). The coding strand (**b**) and the non-coding strand (**c**) were analysed after incubation of *T. reesei* on sophorose (SO), glucose (G) or without carbon source (NC) followed by DMS-induced *in vivo* methylation.

In 1998 the CAE in *T. reesei* was reported to be crucial for regulation of *cbh2* gene expression, encoding a major cellulase (31). Meanwhile, Xyr1 was identified as the major transactivator of most hydrolase-encoding genes including *cbh2* (26,28,43). Allowing one mismatch in the Xyr1-binding motif reveals that the CAE consists of a putative Xyr1-binding site and an overlapping CCAAT-box, which is a common *cis* element in URRs of eukaryotes. Therefore, we analysed an URR including the CAE as well as two additional, *in silico* identified Xyr1-binding sites (allowing one mismatch) and a putative Cre1-binding site (Figure 5a). We performed *in vivo* footprinting of mycelia from repressing conditions (glucose), inducing conditions (sophorose), and used the sample gained from incubation without carbon source as the reference condition.

The CCAAT-box within the CAE and an adjacent A-stretch reacts strongly glucose-dependent (Figure 5c, G/NC, SO/G), while the Xyr1-binding site within the CAE does not yield condition-specific differences (Figure 5b). These two observations might suggest that a carbon source-specific response is mediated via the CCAAT-box, while Xyr1 binds permanently. The latter assumption is in good accordance with the finding that no *de novo* synthesis of Xyr1 is necessary for an initial induction of target genes suggesting that Xyr1 is always available in the cell at a low level (44). However, the new method demonstrates that the two additional, *in silico* identified Xyr1-binding sites are active, but seem to be contacted in a condition-dependent way (Figure 5b and c). This coincides with findings that *cbh2* induction by sophorose goes along with increased *xyr1* transcript formation (45). Finally, the condition-dependent comparison reveals a not yet verified single Cre1-binding site as active regulatory element giving glucose-dependent signals (Figure 5b). Transcript analysis of *cbh2* is complementary to *in vivo* footprinting data, e.g. the induction-dependent (sophorose) protection of the activator’s (Xyr1)-binding sites or the repression-dependent (glucose) protection of the repressor’s (Cre1)-binding site (Supplementary Figure S2b).

#### *In vivo* footprinting of the URR of the *xyn2* gene

**Figure 6.**
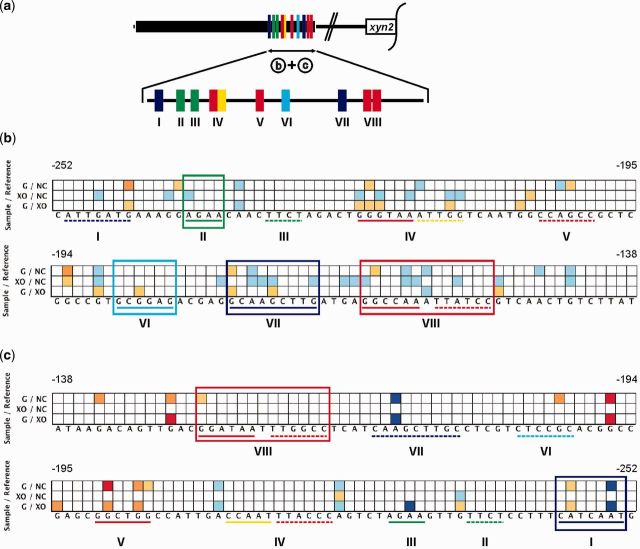
*In vivo* footprinting analysis of the *T. reesei xyn2* URR. (**a**) A *xyn2* URR showing a high number of cis elements [AGAA-box (green), CCAAT-box (yellow), Xyr1-binding site (red) and Cre1-binding site (blue)] was investigated (−252- to −138-bp upstream from ATG). The coding strand (**b**) and the non-coding strand (**c**) were analysed after incubation of *T. reesei* on glucose (G), d-xylose (XO) or without carbon source (NC) followed by DMS-induced *in vivo* methylation. Newly identified motifs are given in frame, motifs with DNA sequence not reported before are given in purple.

The URR of the *xyn2* gene, whose product is the main endo-xylanase of *T. reesei*, has a similar architecture as the one of *cbh2*. In 2003 the XAE comprising a CCAAT-box adjacent to a Xyr1-binding site was reported to be essential for *xyn2* expression (30). The XAE is located close to a second Xyr1-binding site (bearing two mismatches) (Figure 6a, IV, and V). Both Xyr1-binding sites need to be intact for binding Xyr1 *in vitro* and *in vivo* (46). Upstream of the XAE an AGAA-box has before been described as a *cis* element mediating repression (46,47) (Figure 6a, III). We performed *in vivo* footprinting of mycelia from repressing conditions (glucose), inducing conditions (d-xylose), and the reference condition (without carbon source). On the one hand we confirmed the above-mentioned, previously identified *cis* elements, and additionally, revealed condition-dependent contacting by their *trans* factors (Figure 6b and c).

Interestingly, the new *in vivo* footprinting method identified a second AGAA-box, which is located 4-bp upstream of the first one and arranged as inverted repeat (Figure 6b, II). The occurrence of the AGAA-motif as a palindrome is in accordance to an earlier report that this *cis* element is contacted by a basic helix–loop–helix transcription factor, which canonically binds as dimer (47). Also a yet not recognized, single Cre1-binding site could be identified (Figure 6b, VI) exhibiting a glucose-dependent protection (Figure 6b, G/XO; 6 c, G/NC). Additionally, a palindromic Xyr1-binding site spaced by 1 bp was revealed, of which both sites yield condition-specific differences (Figure 6b, c, VIII).

However, *in vivo* footprinting of this region highlighted two more regions, which are contacted in a condition-dependent way. The first one, 5′-ATTGATG-3′ (−251 to −245 bp), yields signals on both investigated strands (Figure 6b, c, I) and bears an unusual TCAAT-box (Figure 6c, I). The second one, 5′-GCAAGCTTG-3′ (−177 to −169 bp), also yields signals on both investigated strands and contains an octameric palindrom (CAAGCTTG) overlapping with an Ace1-binding site [5′-AGGCA-3′, (48)] (Figure 6b, c, VII). Ace1 is a narrow domain transcription factor functioning as repressor of cellulase and xylanase expression (48). A sound interpretation of transcript analysis (Supplementary Figure S2c) compared with *in vivo* footprinting data in this case is difficult to provide because too many new motifs, of which the regulatory function is unknown, were identified. However, induction- or repression-dependent protection/hypersensitivity was observed indicating regulatory functionality.

#### Comparison of regulatory and non-regulatory regions

In order to validate the false positive signal rate of the method we performed footprinting analyses of longer upstream sequences of the above-described genes, i.e. *xyn1*, *xyn2* and *cbh2*. The analysed fragments cover regions previously reported to be regulatory and non-regulatory each (29–31,34,41,49). The heatmaps obtained by referring *in vivo* footprinting results from repressing and inducing conditions to each other are provided in Supplementary Figures S3–S5, respectively. To get additional indication on protein–DNA interaction, the reference of *in vivo* footprinting samples to naked DNA is also included. It is noteworthy that previously identified motifs (Figures 4–6) also show protection/hypersensitivity when *in vivo* footprinting samples were compared with naked DNA (Supplementary Figures S3–S5). Most of the additionally detected signals can be assigned to known motifs (details are described in the respective legends to Supplementary Figures S3–S5), whereas long sequence stretches without any known motif hardly yielded signals. In case of *xyn2,* the two newly identified, before unknown motifs (compare Figure 6, I, VII) are also displayed by the comparison to naked DNA (Supplementary Figure S5).

### Potential of the *in vivo* footprinting method

As already outlined, the software-based *in vivo* footprinting method presented in this study provides the possibility to identify *cis* elements in a fast and sensitive way. We are convinced that this method is a very useful tool for a broad range of investigations concerning regulatory elements, not only in filamentous fungi, but in all organisms. It is important to note that in this study footprinting was performed with DMS followed by HCl DNA cleavage because this a good practice in case of filamentous fungi. However, the proposed approach, in particular DNA fragment analysis and data analysis by ivFAST, is not limited to a certain footprinting or DNA cleavage reagent. The ivFAST manual explains how to adjust parameters in order to analyse data obtained from other footprinting techniques. Compared with the traditional *in vivo* footprinting approach our method employs an internal standard. This allows a comparison of the URR of a gene from any number of conditions or strains, cell lines or tissues without necessity for generating all data at the same time. Because of this and the fact that the method is highly robust (biological and technical replicates do not show relevant differences) it is possible to establish an open-end database for each URR of interest. Furthermore, generated datasets provide a quantitative insight into *trans* factor/*cis* element interaction depicted by a gradual display of results (heatmap). The new footprinting method allows the identification of new variants of already known *cis* elements and of completely new motifs. This is achieved by shuffling the respective, pair-wise comparisons of conditions or cells of interest. Including data from *trans* factor deletion strains/cells in such a database makes the assignment of the according *cis* element possible. It is noteworthy that some improvements of the described technique are also useful for *in vitro* footprinting of purified proteins.

## SUPPLEMENTARY DATA

Supplementary Data are available at NAR Online, including [50,51].

## FUNDING

Austrian Science Fund FWF [P20192 to R.L.M., V232-B20 to A.R.M.-A.]; Vienna University of Technology [AB-Tec doctoral program]; Iogen Corp. (to R.L.M.). Funding for open access charge: Austrian Science Fund FWF.

*Conflict of interest statement*. None declared.

## Supplementary Material

Supplementary Data
