# Supplementary material for: A highly sensitive in vivo footprinting technique for condition-dependent identification of cis elements
Source: Nucleic Acids Res. 2013 Oct 3;42(1):e1. doi: 10.1093/nar/gkt883 (PMC3874196; doi:10.1093/nar/gkt883)
Supplement: Supplementary Data [file supp_42_1_e1__index.html]

A highly sensitive in vivo footprinting technique for condition-dependent identification of cis elements — A highly sensitive in vivo footprinting technique for condition-dependent identification of cis elements — Supplementary Data 

# A highly sensitive *in vivo* footprinting technique for condition-dependent identification of *cis* elements

## Supplementary Data

files

**Files in this Data Supplement:**

- Supplementary Data - pdf file
