# Supplementary material for: A highly sensitive in vivo footprinting technique for condition-dependent identification of cis elements
Source: Nucleic Acids Res. 2013 Oct 3;42(1):e1. doi: 10.1093/nar/gkt883 (PMC3874196; doi:10.1093/nar/gkt883)
Supplement: Supplementary Data [file supp_gkt883_nar-01065-met-g-2013-File008.pdf]

Supplementary figure legends

Supplementary figure S1. An example of a CGE result provided by custom service as *.fsa-file. (a) CGE results of the *cbh2* coding strand following *in vivo* footprinting after replacement on glucose for 3 h (blue), GeneScan 500-ROX internal size standard (red). (b) A zoom into the region of interest including the CAE element is given. The peaks are labelled with the corresponding sequence.

Supplementary figure S2. Analysis of transcript level ratios. *T. reesei* was pre-cultured on glycerol and was thereafter transferred to MA medium without a carbon source (NC), or to MA medium containing glucose (G) as the sole carbon source or to MA media containing sophorose (SO) or D-xylose (XO) as an inducer. Incubation was performed for 3 or 5 h. Transcript levels of the genes *xyn1* (a), *cbh2* (b), and *xyn2* (c) were normalized against *act* and *sar1* transcript levels, and calculated using REST 2009. Transcript level ratios always refer to the sample without carbon source, which is indicated by an asterisk. Results are given in logarithmic scale (lg). The values are means of two independent biologic experiments measured in triplicates. Error bars indicate standard deviations.

Supplementary figure S3. *In vivo* footprinting analysis of the *T. reesei xyn1* 5' upstream region. (a) A *xyn1* fragment covering a non-regulatory region (white bar) and a functional regulatory region (grey bar) bearing five already characterised, functional regulatory elements, *i.e*. a CCAAT-box (yellow), two Xyr1-binding sites (red), and two Cre1-binding sites (blue) (shown in lane SA) was investigated. 5'-UR, 5' upstream region; DA, regulatory region identified by promoter deletion analyses (34). SA, functional binding sites identified by site mutagenesis (29); AF, fragment analyzed during the present study (indicated by a black arrow). (b) The non-coding strand (-270 to -482 bp upstream from ATG) of the *T. reesei* parental strain cultivated on glucose (G) or D-xylose (XO) followed by DMS-induced *in vivo* methylation and naked DNA as a reference (ND) was analyzed. Grey arrow indicates the border between the previously identified non-regulatory and the regulatory region. For comparison to naked DNA the gradual visualization was set as follows: protected bases are highlighted in red shades and hypersensitive bases are highlighted in blue shades; 2.5-fold to 5.0-fold difference between compared conditions is shown in light shaded colour, 5.0-fold to 7.5-fold difference between compared conditions is shown in middle shaded colour, and more than 7.5-fold difference between compared conditions is shown in dark shaded colour. For comparison of *in vivo* footprinting samples the gradual visualisation kept at default settings (see manuscript and legend to main figure 1).
Additionally detected signals could mostly be assigned to putative regulatory elements *i.e.* two Cre1-binding sites (blue), an Ace1-site (orange) (48), and an AbaA-binding element (lavender) previously identified to be responsible for the determination of conidiophore development in *A. nidulans* (50). The latter finding is in good accordance with recent reports on expression of cellulases and hemicellulases as a major event during conidia development in *T. reesei* (51).

Supplementary figure S4. *In vivo* footprinting analysis of the *T. reesei cbh2* 5' upstream region. (a) A *cbh2* fragment covering a non-regulatory region (white bar) and a functional regulatory region (grey bar) bearing two already characterised, functional regulatory elements, *i.e.* a CCAAT-box (yellow) and

a Xyr1-binding site (red) (shown in lane SA) was investigated. 5’-UR, 5’ upstream region; DA, regulatory region identified by promoter deletion analyses (49), EMSA, region involved in DNA-protein complex formation according to EMSA analyses (49); SA, functional binding sites identified by site mutagenesis (31); AF, fragment analyzed during the present study (indicated by a black arrow). (b) The coding strand (-263 to -148 bp upstream from ATG) of the *T. reesei* parental strain cultivated on glucose (G) or sophorose (SO) followed by DMS-induced *in vivo* methylation and naked DNA as a reference (ND) was analyzed. Grey arrows indicate the start and the end of the previously identified regulatory region. For settings of the gradual visualization see legend to Fig. S3.
Additionally detected signals could be assigned to putative regulatory elements, *i.e.* three Xyr1-binding sites and one Cre1-binding sites (blue).

Supplementary figure S5. *In vivo* footprinting analysis of the *T. reesei xyn2* 5’ upstream region. (a) A *xyn2* fragment covering a non-regulatory region (white bar) and a functional regulatory region (grey bar) bearing four already characterised, functional regulatory elements, *i.e.* one AGAA-box (green), two Xyr1-binding sites (red), and a CCAAT-box (yellow) (shown in lane SA) was investigated. 5’-UR, 5’ upstream region; DA, regulatory region identified by promoter deletion analyses (41); SA, functional binding sites identified by site mutagenesis (30,46); AF, analyzed during the present study (indicated by a black arrow). (b) The coding strand (-260 to -82 bp upstream from ATG) of the *T. reesei* parental strain cultivated on glucose (G) or D-xylose (XO) followed by DMS-induced *in vivo* methylation and naked DNA as a reference (ND) was analyzed. Grey arrows indicate the start and the end of the previously identified regulatory region. For settings of the gradual visualization see legend to Fig. S3.
Additionally detected signals could mostly be assigned to putative regulatory elements, *i.e.* two Xyr1 sites, a Cre1-binding site (blue). A putative TATA-box (black) yielded strong signals. The two newly identified motives (purple) could be detected, one of which bearing an unusual TCAAT-box, the other represents an octameric palindrome overlapping with an Ace1-site (48).

Supplementary references

29. Rauscher, R., Würleitner, E., Wacenovsky, C., Aro, N., Stricker, A.R., Zeilinger, S., Kubicek, C.P., Penttilä, M. and Mach, R.L. (2006) Transcriptional regulation of *xyn1*, encoding xylanase I, in *Hypocrea jecorina*. *Eukaryot Cell*, **5**, 447-456.

30. Würleitner, E., Pera, L., Wacenovsky, C., Cziferszky, A., Zeilinger, S., Kubicek, C.P. and Mach, R.L. (2003) Transcriptional regulation of *xyn2* in *Hypocrea jecorina*. *Eukaryot Cell*, **2**, 150-158.

31. Zeilinger, S., Mach, R.L. and Kubicek, C.P. (1998) Two adjacent protein binding motifs in the *cbh2* (cellobiohydrolase II-encoding) promoter of the fungus *Hypocrea jecorina* (*Trichoderma reesei*) cooperate in the induction by cellulose. *J Biol Chem*, **273**, 34463-34471.

34. Mach, R.L., Strauss, J., Zeilinger, S., Schindler, M. and Kubicek, C.P. (1996) Carbon catabolite repression of xylanase I (*xyn1*) gene expression in *Trichoderma reesei*. *Mol Microbiol*, **21**, 1273-1281.

41. Zeilinger, S., Mach, R.L., Schindler, M., Herzog, P. and Kubicek, C.P. (1996) Different inducibility of expression of the two xylanase genes *xyn1* and *xyn2* in *Trichoderma reesei*. *J Biol Chem*, **271**, 25624-25629.

46. Stricker, A.R., Trefflinger, P., Aro, N., Penttilä, M. and Mach, R.L. (2008) Role of Ace2 (Activator of Cellulases 2) within the *xyn2* transcriptosome of *Hypocrea jecorina*. *Fungal Genet Biol*, **45**, 436-445.

48. Aro, N., Ilmén, M., Saloheimo, A. and Penttilä, M. (2003) ACEI of *Trichoderma reesei* is a repressor of cellulase and xylanase expression. *Appl Environ Microbiol*, **69**, 56-65.

49. Stangl, H., Gruber, F. and Kubicek, C.P. (1993) Characterization of the *Trichoderma reesei cbh2* promoter. *Curr Genet*, **23**, 115-122.

50. Andrianopoulos, A. and Timberlake, W.E. (1994) The *Aspergillus nidulans abaA* gene encodes a transcriptional activator that acts as a genetic switch to control development. *Mol Cell Biol*, **14**, 2503-2515.

51. Metz, B., Seidl-Seiboth, V., Haarmann, T., Kopchinskiy, A., Lorenz, P., Seiboth, B. and Kubicek, C.P. (2011) Expression of biomass-degrading enzymes is a major event during conidium development in *Trichoderma reesei*. *Eukaryot Cell*, **10**, 1527-1535.

Fig. S1

a

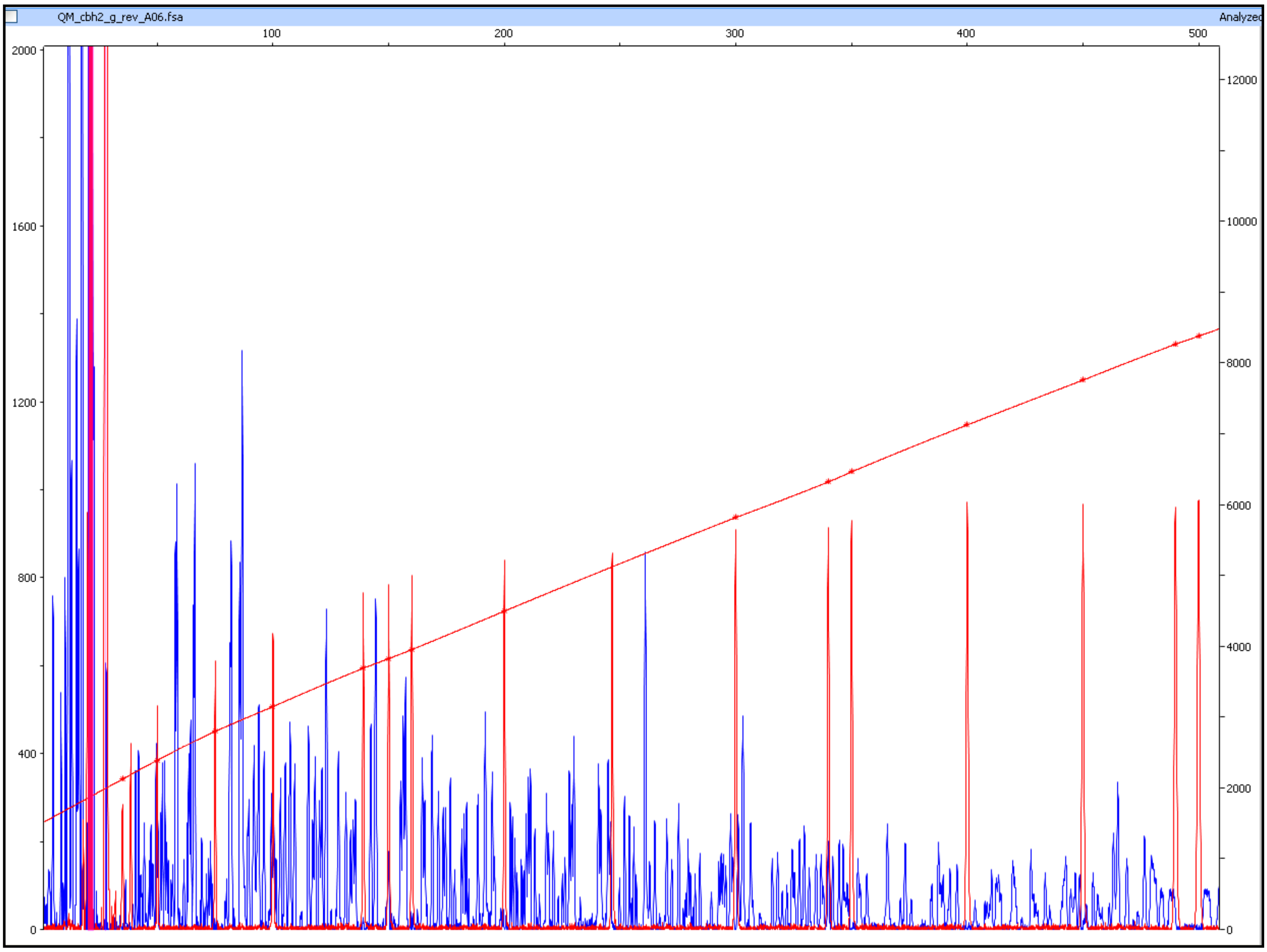

b

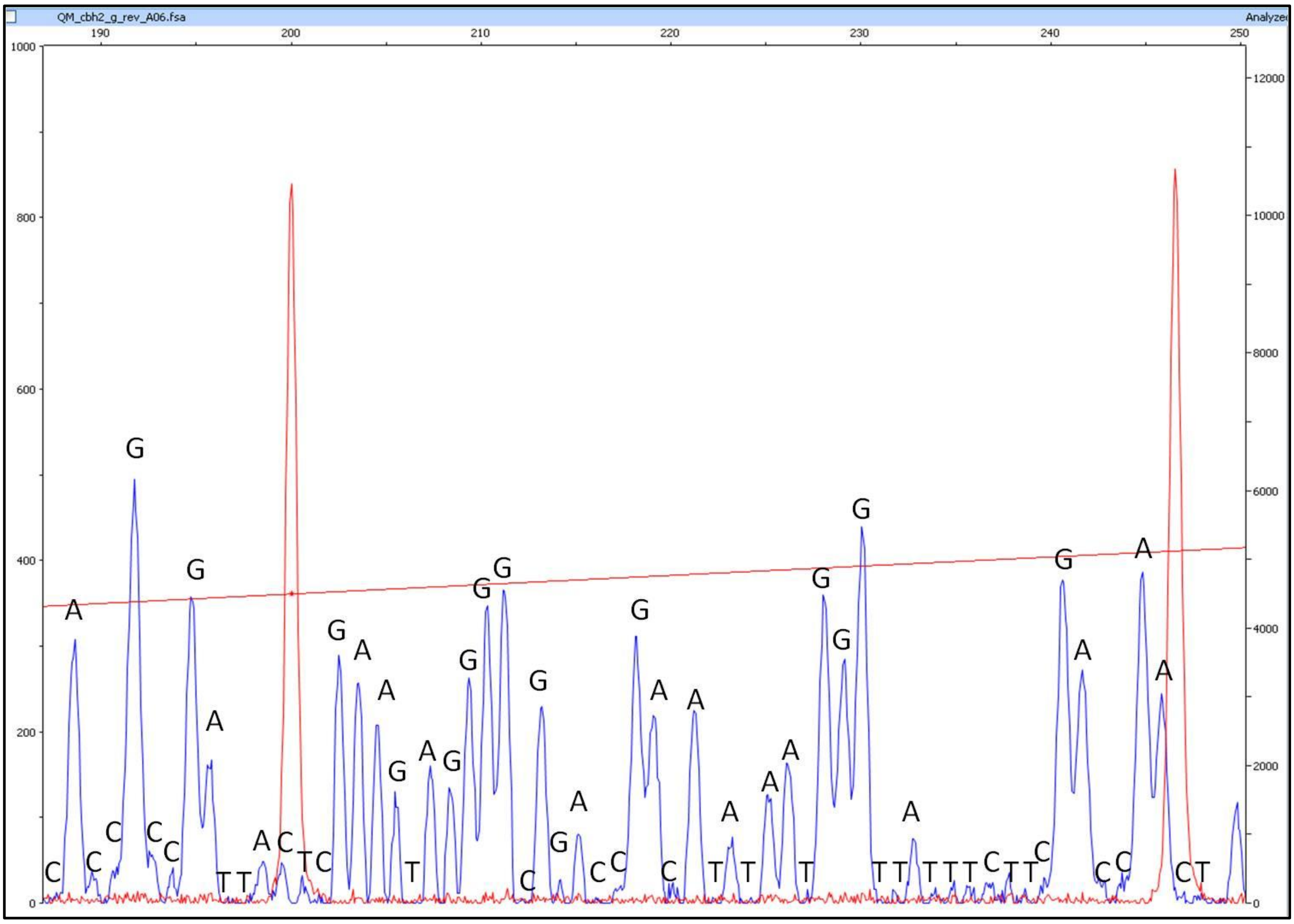

Fig. S2

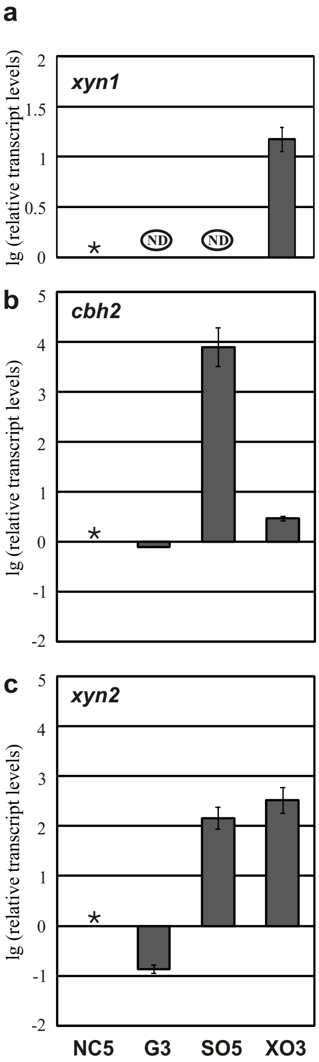
a
xyn1
lg (relative transcript levels)
2
1.5
1
0.5
0
*
ND
ND
b
cbh2
lg (relative transcript levels)
5
4
3
2
1
0
-1
-2
*
c
xyn2
lg (relative transcript levels)
5
4
3
2
1
0
-1
-2
*
NC5
G3
SO5
XO3

Fig. S3

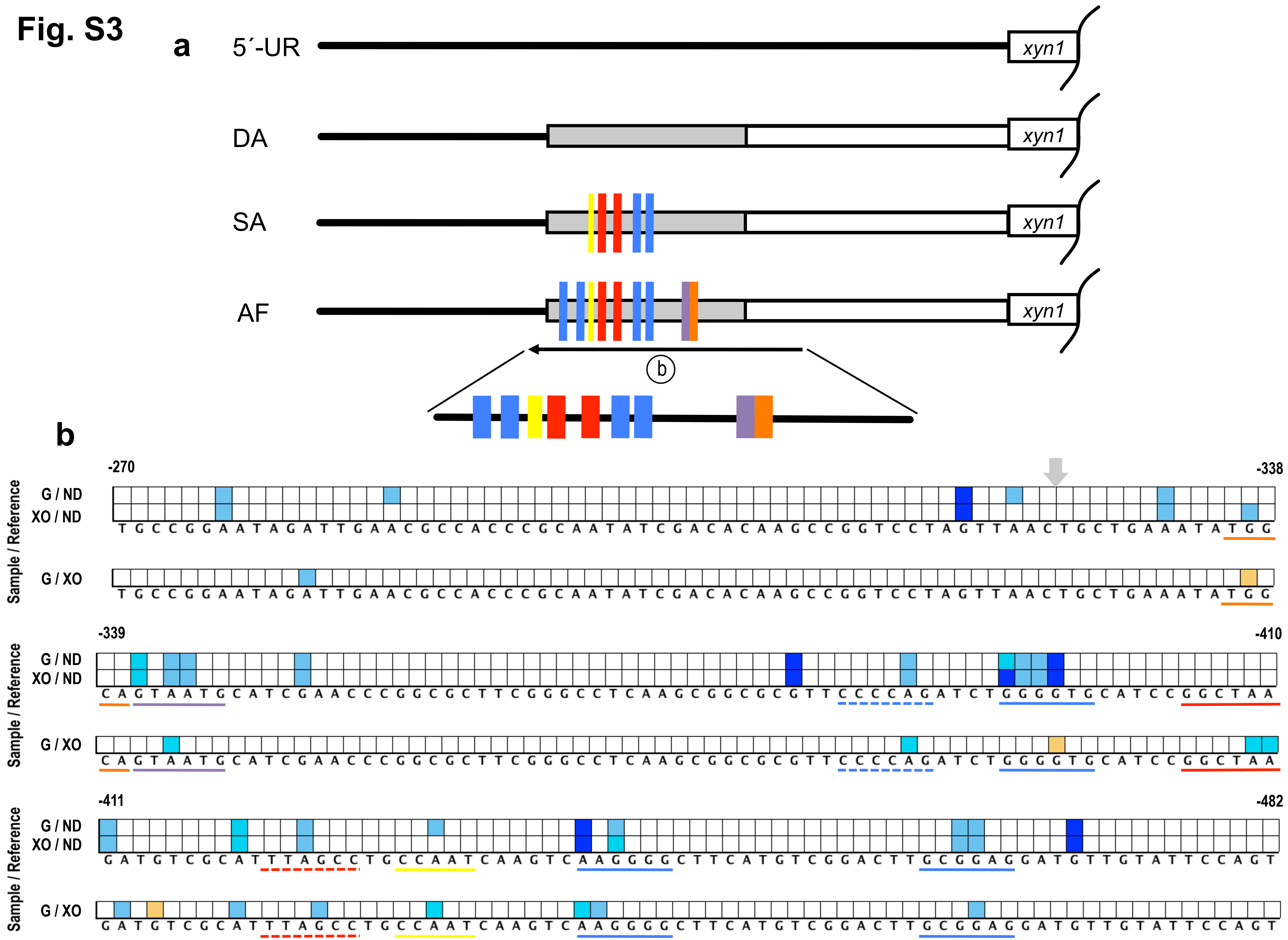

a
5´-UR
xyn1
DA
SA
AF
b
b
-270
-338
G / ND
XO / ND
T G C C G G A A T A G A T T G A A C G C C A C C C G C A A T A T C G A C A C A A G C C G G T C C T A G T T A A C T G C T G A A A T A T G G
G / XO
T G C C G G A A T A G A T T G A A C G C C A C C C G C A A T A T C G A C A C A A G C C G G T C C T A G T T A A C T G C T G A A A T A T G G
-339
-410
G / ND
XO / ND
C A G T A A T G C A T C G A A C C C G G C G C T T C G G G C C T C A A G C G G C G C G T T C C C C A G A T C T G G G G T G C A T C C G G C T A A
G / XO
C A G T A A T G C A T C G A A C C C G G C G C T T C G G G C C T C A A G C G G C G C G T T C C C C A G A T C T G G G G T G C A T C C G G C T A A
-411
-482
G / ND
XO / ND
G A T G T C G C A T T T A G C C T G C C A A T C A A G T C A A G G G G C T T C A T G T C G G A C T T G C G G A G G A T G T T G T A T T C C A G T
G / XO
G A T G T C G C A T T T A G C C T G C C A A T C A A G T C A A G G G G C T T C A T G T C G G A C T T G C G G A G G A T G T T G T A T T C C A G T
Sample / Reference

Fig. S4

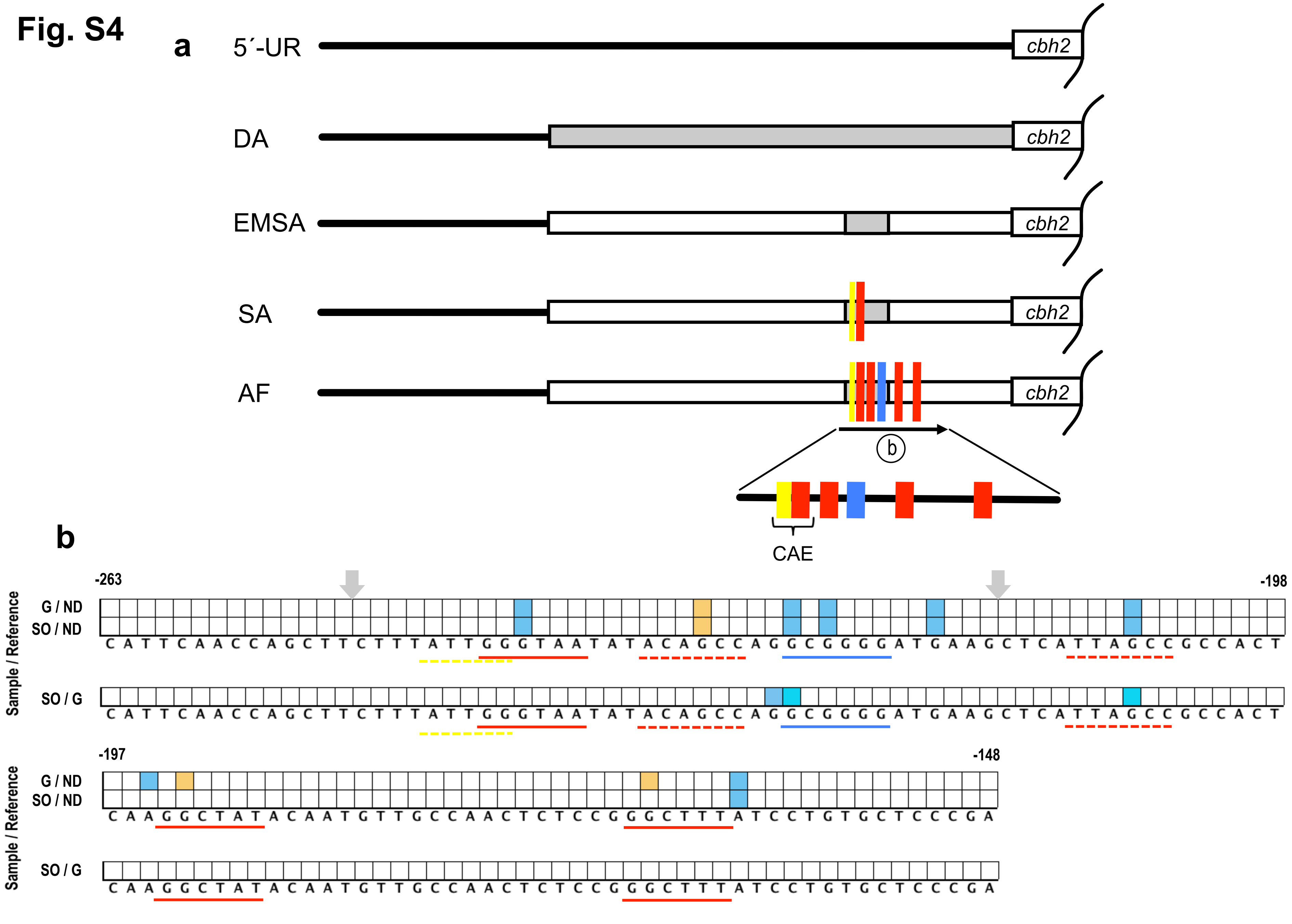
a
5´-UR
DA
EMSA
SA
AF
cbh2
b
CAE
b
-263
-198
G / ND
SO / ND
SO / G
Sample / Reference
C A T T C A A C C A G C T T C T T T A T T G G G T A A T A T A C A G C C A G G C G G G A T G A A G C T C A T T A G C C G C C A C T
-197
-148
C A A G G C T A T A C A A T G T T G C C A A C T C T C C G G G C T T T A T C C T G T G C T C C C G A

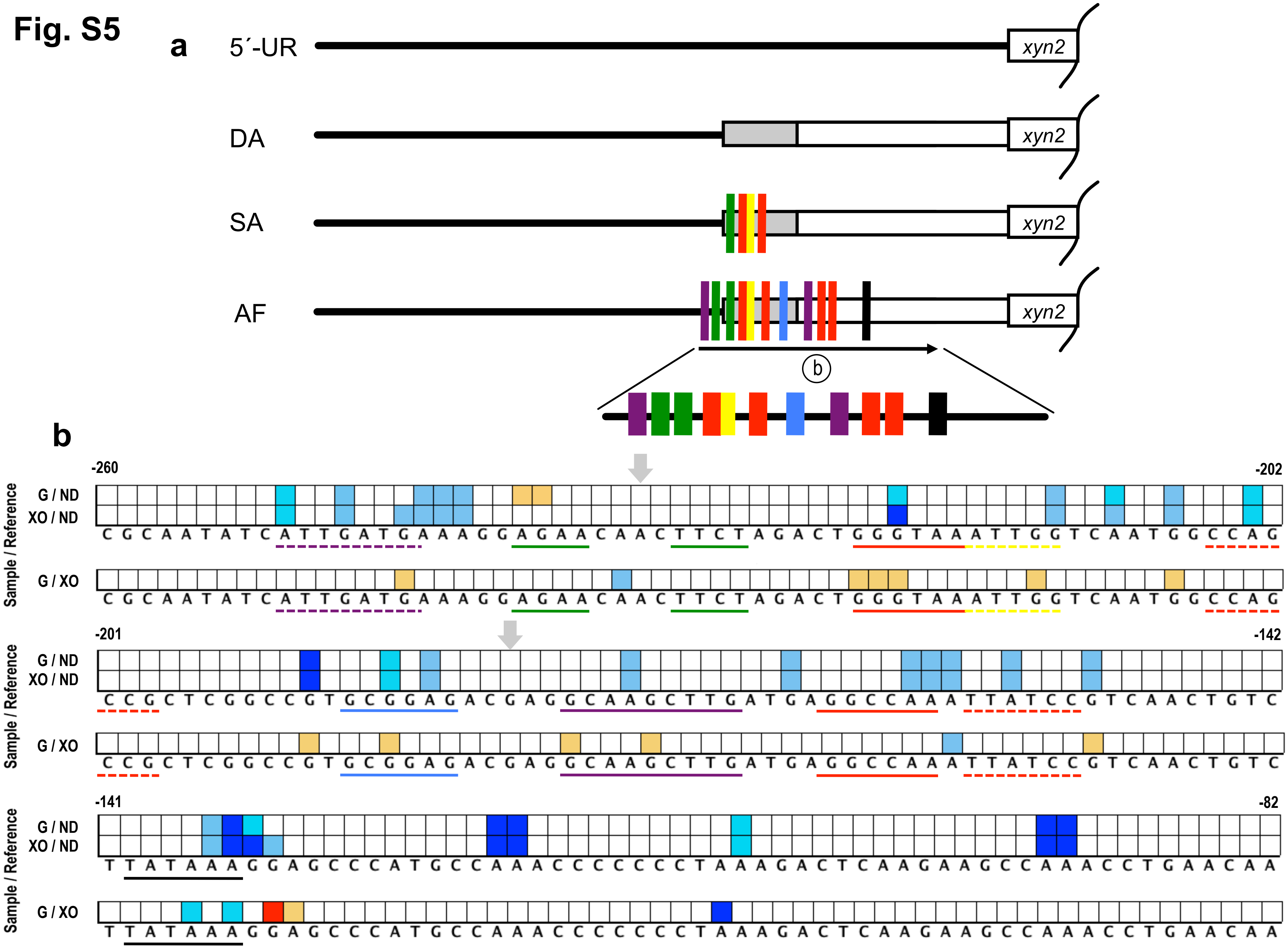

Fig. S5
a
5´-UR
DA
SA
AF
xyn2
b
-260
-202
G / ND
XO / ND
G / XO
C G C A A T A T C A T T G A T G A A A G G A G A A C A A C T T C T A G A C T G G G T A A A T T G G T C A A T G G C C A G
-201
-142
C C G C T C G G C C G T G C G G A G A C G A G G C A A G C T T G A T G A G G C C A A A T T A T C C G T C A A C T G T C
-141
-82
T T A T A A A G G A G C C C A T G C C A A A C C C C C C C T A A A G A C T C A A G A A G C C A A A C C T G A A C A A
Sample / Reference
